# Time and risk factors for death among smear-positive pulmonary tuberculosis patients in the Health District of commune VI of Bamako, Mali, 2016

**DOI:** 10.1186/s12889-021-10986-4

**Published:** 2021-05-18

**Authors:** Yaya Ballayira, Pauline Kiswendsida Yanogo, Bakary Konaté, Fadima Diallo, Bernard Sawadogo, Simon Antara, Nicolas Méda

**Affiliations:** 1 Burkina Field Epidemiology Training Program, University Joseph KI-ZERBO, Ouagadougou, Burkina Faso; 2 Department of Public Health, Faculty of medicine, University Joseph KI-ZERBO, Ouagadougou, Burkina Faso; 3 National Directorate of Health, Ministry of Health, Bamako, Mali; 4 African Field Epidemiology Network, Kampala, Uganda

## Abstract

**Background:**

The End Tuberculosis (TB) Strategy aims to achieve 90% reduction of deaths due to TB by 2030, compared with 2015. Mortality due to tuberculosis in Mali was 13 per 100,000 inhabitants in 2014 and 11 per 100,000 inhabitants in 2017. Risk factors for death are not known. The objective of this study was to determine the time and risk factors for death in pulmonary TB patients with positive microscopy.

**Methods:**

We conducted a retrospective cohort study from October to December 2016 in Commune VI of Bamako. Smear positive cases pulmonary tuberculosis from 2011 to 2015 were included. We reviewed the treatment registers and collected sociodemographic, clinical, biological and therapeutic data. Median time to death and hazard ratio (HR) were estimated by the Kaplan-Meier method and a Cox regression model, respectively.

**Results:**

In total, we analysed 1362 smear positive cases of pulmonary TB including 104 (8%) HIV positive and 90 (7%) deaths. The mean age was 36 ± 13 years, the sex ratio of males to females was 2:1. Among the deaths, 48 (53%) occurred during the first 2 months of treatment. Age ≥ 45 years (HR 2.09 95% CI [1.35–3.23]), weight <  40 kg (HR 2.20 95% CI [1.89–5.42]), HIV unknown status (HR 1.96, 95% CI [1.04–3.67]) and HIV-positive (HR 7.10 95% CI [3.53–14.26]) were significantly associated with death.

**Conclusions:**

The median time to death was 2 months from the start of treatment. Independent risk factors for death were age ≥ 45 years, weight <  40 kg, unknown and positive HIV status. We recommend close monitoring of patients over 45 years, HIV testing in those with unknown status, an adequate care for positive HIV status, as well as a nutritional support for those with weight below 40 kg during the intensive phase of TB treatment.

## Background

The Global Tuberculosis (TB) report estimated that 10.0 million people developed TB disease in 2017, with 1.3 million deaths among HIV-negative people and with an additional 300,000 deaths from TB among HIV-positive people [1]. The new End TB Strategy aims to reduce by 95% the deaths due to tuberculosis and by 90% the tuberculosis incidence rate by 2035 compared with 2015 [2]. Identification of the factors associated with dying from TB is useful for evidence-based interventions to achieve these objectives. In Mali, TB mortality was 13 per 100,000 population in 2014, and 11 per 100,000 in 2017 [1, 3], a relative decrease of 15%. To this evolution, the objectives of the End TB strategy may not be achieved in Mali in 2035.

In 2009, a study conducted in Commune VI of Bamako found a lethality of 14% among cases of smear positive pulmonary tuberculosis (SPPTB) [4]. However, to our knowledge, no study has been conducted to identify the risk factors for death from SPPTB in this district. In the literature, these deaths occurred often in the first 2 months of treatment [5–8]. Many factors could explain this.

Older age [5, 9, 10], male sex [7, 8, 11, 12], unemployment [13], imprisonment [7] have been associated with death in several studies. In a study in South Korea, alcohol consumption several times a week was identified as a protective factor [14]. However, in a systematic review alcohol abuse was a risk factor for death from SPPTB [15]. In other studies, low weight [5, 16], TB / HIV coinfection [5, 6, 12, 13, 17], cases of tuberculosis retirees were at higher risk of dying during treatment [9, 12, 14]. Our aim was to determine the time and the risk factors for death in positive microscopy pulmonary tuberculosis in the health district of Commune VI of Bamako for treatment cohorts from 2011 to 2015.

## Methods

### Type and populations of study

A retrospective cohort study was conducted from October 20, 2016 to December 30, 2016. Data from 2011 to 2015 of SPPTB cases, aged 18 years and older and with treatment outcome known during our study period were collected.

To constitute our sample, we considered all SPPTB in the district of the Commune VI, followed for 6 to 8 months according to the therapeutic categories. Patients under the age of 18 and those without the reported interest variables were excluded. In terms of tuberculosis management, the district has a Diagnostic and Treatment Center, which is the referral health center, and 11 treatment centers which are the community health centers. These centers provide directly observed treatment for tuberculosis patients, a strategy adopted by Mali since 1994.

### Data collected

The variables of interest were those already included in the records for follow-up and treatment of tuberculosis patients in Mali. We collected on sociodemographic (age, sex, residence), clinical (treatment center, initial weight, SPPTB type (new, old case), HIV status (negative, unknown, positive)) therapeutic (treatment category (CAT1, CAT2), treatment dosage, Directly Observed Treatment (DOT), duration of treatment, treatment outcome) and biological (initial smear density) characteristics.

To extract the data, the investigators sorted out the follow-up and processing records from 2011 to 2015. The SPPTB cases were collected and listed on the data extraction form. A comparison was made between the two data sources for each SPPTB case to complete and validate the data.

**Fig. 1:**
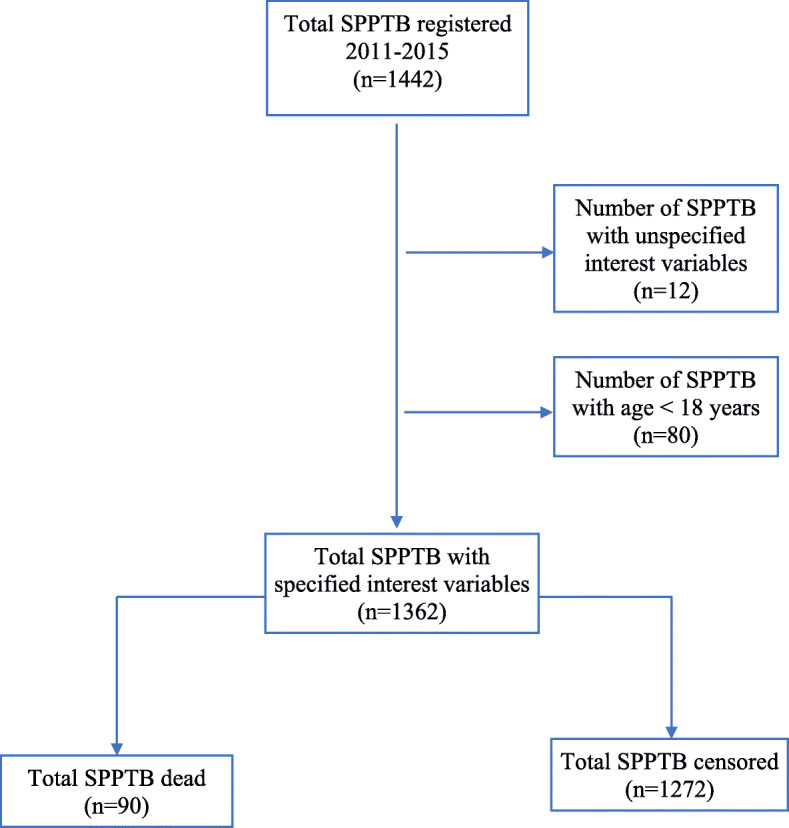
Flowchart representing the process of selecting the study sample

A total of 1362 cases, **(**Fig. 1**)**, including 90 (7%) deaths and SPPTB cases with other outcomes (cured, completed treatment, treatment failures, transferred, lost to follow up) were analyzed.

We defined time-to-death as the time between the initiation of anti-tuberculosis treatment and the occurrence of death. Treatment outcomes (cured, treatment completed, treatment failure, lost to follow-up, unassessed, death) were defined according to WHO criteria [22]. Death is defined as a TB patient who dies for any reason during anti-tuberculosis treatment.

### Statistical analysis

We analyzed data using R version 3.3.1 and Epi Info 7. For descriptive purpose, we estimated, frequencies, proportions, mean and standard deviations. The time-to-death and factors affecting survival were estimated by the Kaplan-Meier method and tested by the Log Rank test. The assumption of proportional hazards was checked and then a univariate Cox proportional hazard models was used to select variables with a *p*-value of < 0.25 for inclusion in the baseline model in multivariate analysis. In a top-down multivariate Cox regression model, we calculated the adjusted Hazard ratio (aHR) that was tested by the Wald test to identify independent risk factors for death in SPPTB in Commune VI of Bamako and a *p-value* < 0.05 was used as significance threshold.

## Results

### General description of the cohort

A total of 1362 cases, **(**Fig. 1**)**, including 90 (7%) deaths and censored SPPTB (curee, completed treatment, failures, transferred, abandoned) were analyzed. The average age was 36 ± 13 years (18–105 years). The male sex represented 950 (70%) cases. There were 1277 (94%) cases whose weight ≥ 40 kg, with an average weight of 53 ± 10 kg (28–105 kg). New cases of SPPTB were 1235 (91%) cases. There were 104 (8%) positive HIV + SPPTB and 846 (62%) unknown HIV cases. Tuberculous patients with a smear density at 3+ on microscopy were 770 (57%) cases. The case fatality rate was 12 deaths per 1000 person-months.

### The intensive phase of treatment as death time

In SPPTB, 48 (53%) of deaths occurred during the first 2 months of TB treatment, with a median time- to-death of 2 months **(**Table 1**).**

**Table 1:** Time to Death of SPPTB under treatment, Commune VI of Bamako, Mali, 2011–2015

Survival time (months)	Number at risk	Deaths	Proportion of deaths (%)
0–1	1362	31	34
1–2	1317	17	19
2–3	1286	16	18
3–4	1255	10	11
4–5	1221	7	8
5–6	1186	8	9
6–7	42	1	1
7–8	30	0	0

### Factors affecting survival time

**Fig. 2:**
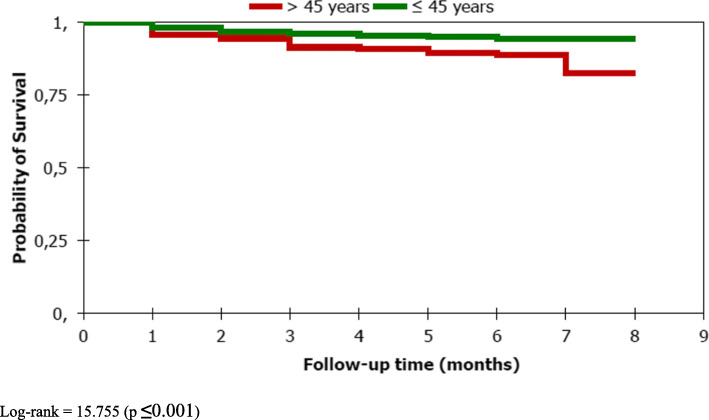
Survival curve of SPPTB cases under treatment by age group, Commune VI of Bamako, Mali, 2011–2015. Log-rank = 15.755 (*p* ≤ 0.001)

**Fig. 3:**
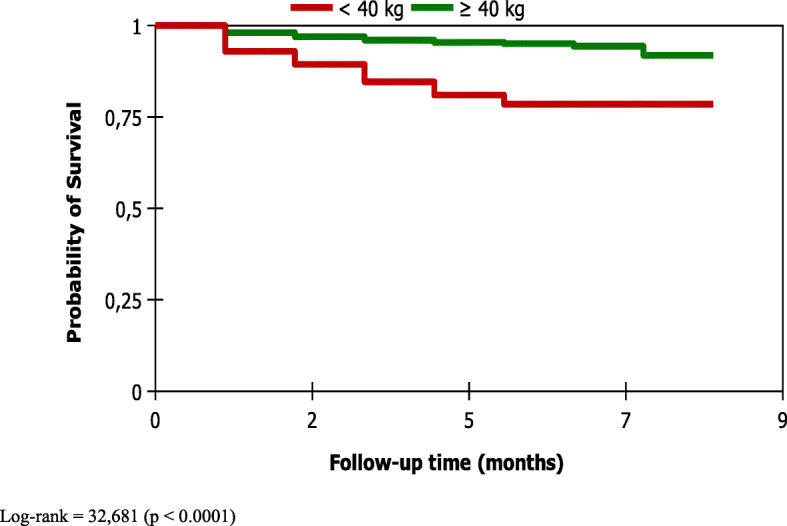
Survival curve of SPPTB cases under treatment by initial weight, Commune VI of Bamako, Mali, 2011–2015. Log-rank = 32,681 (*p* < 0.0001)

**Fig. 4:**
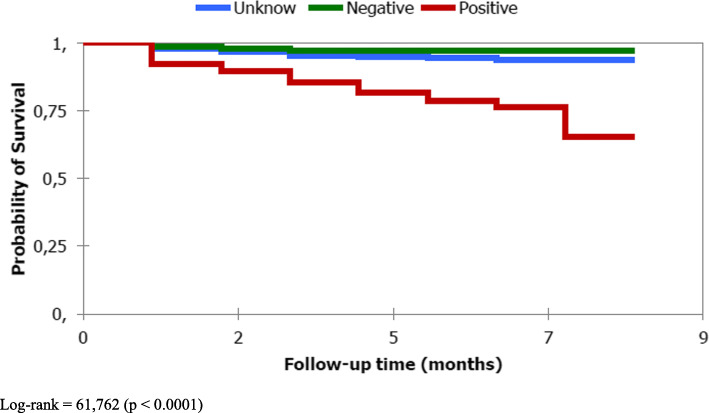
Survival curve of SPPTB under treatment by VIH status, Commune VI of Bamako, Mali, 2011–2015. Log-rank = 61,762 (*p* < 0.0001)

In SPPTB, survival time on the Kaplan Meier curve was significantly different between the younger (< 45 years and older (> 45 years) (*p* **≤** 0.001) **(**Fig. 2**)**, sex (*p* = 0.022), initial weight (*p* < 0.0001) **(**Fig. 3**)** and HIV serology status (*p* < 0.0001) **(**Fig. 4**).** However, the survival time was not significantly different between new and old cases of the SPPTB type (*p* = 0.992), never between the different smear densities (*p* = 0.250).

### Risk factors

In univariate analysis, age ≥ 45 years (HR 2.16; 95% CI [1.40–3.33], *p* **≤** 0.001) male sex (HR 0.61; 95% CI [0.40–0.93] *p* = 0.023), the initial weight <  40 kg (HR 4.06; 95% CI [2.42–6.81], *p* **≤** 0.001), positive HIV status (HR 8.97; 95% CI [4.50–17.86, *p* **≤** 0.001) and unknown (HR 2.18; 95% CI [1.17–4.09], *p* = 0.015) were the factors significantly associated with death in SPPTB **(**Table 2**).**

**Table 2:** Risk Factors for Death among SPPTB in Commune VI of Bamako in Univariate Cox, Mali, 2011–2015

Participants characteristics		SPPTB deaths		HR^i^ [95% CI^**ii**^]	P-value
		Yes	No		
**Age**	< 45 years of age	58	1023	1	-
	≥ 45 years old	32	249	2.16 [1.40–3.33]	**≤**0.001
**Sex**	Female	37	375	1	–
	Male	53	897	0.61 [0.40–0.93]	0.023
**Initial weight**	≥ 40 kg	72	1205	1	–
	< 40 kg	18	67	4.06 [2.42–6.81]	**≤**0.001
**Type of** SPPTB	New case^i^	81	1154	1	–
	Old case^ii^	9	118	1.00 [0.50–2.03]	0.992
**HIV**	Negative	12	400	1	**≤**0.001
	Unknown	53	793	2.18 [1.17–4.09]	
	Positive	25	79	8.97 [4.50–17.86]	
**Smear density**	1+	27	325	1	**0.25**
	2+	10	230	0,55 [0.27–1,14]	
	3+	53	717	0.92 [0.57–1.46]	
**Treatment center**	Semi-rural	59	823	1	–
	Urbain	31	449	0.96 [0.62–1.49]	0.865

^i^New case: never been treated for TB or have taken anti-TB drugs for less than 1 month

^ii^Old case: have received 1 month or more of anti-TB drugs in the past

In multivariate analysis, age ≥ 45 years (HR 2.09 95% CI [1.35–3.23]; *p* **≤** 0.001), weight < 40 kg (HR 2.20 95% CI [1.89–5, 42], *p* **≤** 0.001), HIV status (unknown HIV status (HR 1.96, 95% CI [1.04–4.67], *p* = 0.037) and positive HIV status (HR 7.10 95% CI [3.53–14.26], *p* **≤** 0.001) were the factors independently associated with death in SPPTB **(**Table 3**).**

**Table 3:** Risk Factors for Death among SPPTB in Commune VI of Bamako in Multivariate Analysis, Mali, 2011–2015

Participants characteristics		HR	5%CI	***P***
**Age**	< 45 years of age	1		
	≥ 45 years old	2.09	[1.35–3.23]	**≤**0.001
**Sex**	Female		1	
	Male	1.05	[0.65–1.69]	0.840
**Initial weight**	≥ 40 kg		1	
	< 40 kg	2.20	[1.89–5.42]	**≤**0.001
**HIV**	Negative	1		
	Unknown	1.96	[1.04–3.67]	0.037
	Positive	7.10	[3.53–14.26]	**≤**0.001

## Discussion

Our study found that the majority of deaths (53%) occurred during the intensive phase of TB treatment. This is consistent with other studies in different countries [5, 7, 18].

The factor most strongly associated with death in our study was positive HIV status. SPPTB / HIV positive patients were 7 times more likely to die during TB treatment than SPPTB / HIV negative. This is concordant with a systematic review that found that in the context of high incidence of tuberculosis and HIV seroprevalence, HIV was found to be a risk factor, but not in areas with low tuberculosis incidence and HIV seroprevalence [17]. However, early initiation of TB / HIV patients on ARV and cotrimoxazole therapy may decrease this lethality. Data on ARV and cotrimoxazole use were not included in our study. These data would probably improve the current analyses. In Nigeria, TB/HIV coinfection, not receiving ARVs, not receiving cotrimoxazole were independently associated with death of TB patients [6]. Other studies have also found an association between HIV and death among TB patients [5, 8, 9, 12, 14, 19, 20]. Vasankari in Finland found immunosuppression, virtually all due to causes other than HIV infection, and concomitant diseases and their medical treatment to be a risk factor for death of TB patients [11].. Only two patients in his cohort had HIV-coinfection. The second most important factor was the weight < 40 kg at the initiation of treatment. We found that SPPTB patients whose initial weight was below 40 kg were 3 times more likely to die and this finding is similar to those found in studies conducted in different countries [5, 14, 16]. Malnutrition, which can lead to underweight, has been one of the risk factors for death among tuberculosis patients [17]. The third most important factor was age ≥ 45 years. Patients with aged 45 years or older had twice the instant risk of dying during tuberculosis treatment.

This is also supported by many other studies conducted in Africa [5, 8, 9, 12], in Europe [11] and in Asia [10, 13–15]. The last factor associated was unknown HIV status. SPPTB patients with unknown HIV status had 2 times more instant risk of dying on TB treatment than those with negative / HIV status. Patients with unknown HIV status may contain both HIV positive and HIV negative cases. Given the difference in risk of death compared to HIV negative, there may be enough HIV positive cases to achieve this result. Testing for HIV would give us more edification. Similarly, a study conducted in South Africa, has found unknown HIV status had twice as much risk of dying compared to negative HIV status [9].

This study has some limitations. As our study was based on surveillance data, some informations were not collected which did not allow us to test and adjust on other factors such as unemployment status, alcohol abuse, anemia, rural dwelling and comorbidities (heart disease, end-stage renal disease, diabetes, cancer) that have been considered as risk factors in the literature. However, this work has some strengths. To our knowledge, this study is the first publication to study the time and risk factors for death in patients with positive microscopy pulmonary tuberculosis in the District of Commune VI of Bamako. The study covered a period of 5 years and concerned all patients in the district; providing a good representativeness for the estimation of the factors sought.

## Conclusions

The majority of deaths among SPPTB patients on treatment occurred during the first two months of treatment which is the intensive phase of the TB treatment course. Older age (≥ 45 years), low weight at treatment initiation (< 40 kg), unknown and positive HIV status were the independent risk factors associated with SPPTB deaths. We recommend that the National Tuberculosis Program establish a specific system to closely monitor SPPTB patients aged 45 years or older and patients with positive or unknown HIV status, also screen for HIV in patients with unknown HIV status. We also recommend nutritional support for malnourished SPPTB patients.

## Data Availability

Data are available on request to the following authors (Ballayira Yaya and Yanogo Pauline Kiswendsida).
